# Rationing of nursing care interventions and its association with nurse-reported outcomes in the neonatal intensive care unit: a cross-sectional survey

**DOI:** 10.1186/s12912-016-0169-z

**Published:** 2016-08-02

**Authors:** Christian M. Rochefort, Bailey A. Rathwell, Sean P. Clarke

**Affiliations:** 1 School of Nursing, Faculty of Medicine, University of Sherbrooke, Campus Longueuil, 150 Place Charles-LeMoyne, Room 200, Longueuil, Quebec J4K 0A8 Canada; 2 Centre de recherche, Hôpital Charles-LeMoyne, 150 Place Charles-LeMoyne, Room 200, Longueuil, Quebec J4K 0A8 Canada; 3 Department of Epidemiology, Biostatistics and Occupational Health, Faculty of Medicine, McGill University, Purvis Hall, 1020 Pine Avenue West, Montreal, Quebec H3A 1A2 Canada; 4 Ingram School of Nursing, McGill University, Wilson Hall, 3506 University Street, Montreal, Quebec H3A 2A7 Canada; 5 William F. Connell School of Nursing, Maloney Hall, Room 218, 140 Commonwealth Avenue, Chestnut Hill, MA 02467 USA; 6 McGill University Health Centre, Montréal, Canada

**Keywords:** Care rationing, Comfort care, Cross-sectional survey, Donabedian, Neonatal Extent of Work Rationing Instrument, Neonatal intensive care unit, Neonatal pain, Readiness for hospital discharge, Registered nurses

## Abstract

**Background:**

Evidence internationally suggests that staffing constraints and non-supportive work environments result in the rationing of nursing interventions (that is, limiting or omitting interventions for particular patients), which in turn may influence patient outcomes. In the neonatal intensive care unit (NICU), preliminary studies have found that discharge preparation and infant comfort care are among the most frequently rationed nursing interventions. However, it is unknown if the rationing of discharge preparation is related to lower perceptions of parent and infant readiness for NICU discharge, and if reports of increased rationing of infant comfort care are related to lower levels of perceived neonatal pain control. The purpose of this study was to assess these relationships.

**Methods:**

In late 2014, a cross-sectional survey was mailed to 285 Registered Nurses (RNs) working in one of 7 NICUs in the province of Quebec (Canada). The survey contained validated measures of care rationing, parent and infant readiness for discharge, and pain control, as well as items measuring RNs’ characteristics. Multivariate regression was used to examine the association between care rationing, readiness for discharge and pain control, while adjusting for RNs’ characteristics and clustering within NICUs.

**Results:**

Overall, 125 RNs completed the survey; a 44.0 % response rate. Among the respondents, 28.0 and 40.0 % reported rationing discharge preparation and infant comfort care “often” or “very often”, respectively. Additionally, 15.2 % of respondents felt parents and infants were underprepared for NICU discharge, and 54.4 % felt that pain was not well managed on their unit. In multivariate analyses, the rationing of discharge preparation was negatively related to RNs’ perceptions of parent and infant readiness for discharge, while reports of rationing of parental support and teaching and infant comfort care were associated with less favourable perceptions of neonatal pain control.

**Conclusions:**

The rationing of nursing interventions appears to influence parent and infant readiness for discharge, as well as pain control in NICUs. Future investigations, in neonatal nursing care as well as in other nursing specialties, should address objectively measured patient outcomes (such as objective pain assessments and post-discharge outcomes assessed through administrative data).

## Background

Numerous studies internationally have provided evidence that nurse understaffing and non-supportive work environments are related to adverse patient outcomes, such as increased morbidity, mortality and costs [1–4]. One proposed mechanism for these findings has been that unfavourable working conditions lead to the rationing of nursing care interventions [5–7]. Care rationing refers to “the withholding of or failure to carry out necessary nursing interventions for patients due to a lack of nursing resources such as staffing, skill mix, or time” [8]. When resources are insufficient, nurses are thus forced to ration their attention across patients and use their clinical judgment to prioritize assessments and interventions; leading to limits on interventions or omissions of aspects of care that may increase the risk of negative patient outcomes [9]. Most research on care rationing to date has focussed on adult settings, with scant attention given to other clinical areas, such as the neonatal intensive care unit (NICU).

Staff in NICUs provide care for the most critically ill infants and nurses on these units are assigned to small numbers of patients [10]. Optimal management of premature infants and their families thus requires a sufficient supply of highly skilled nurses and supportive work environments [11, 12]. Despite these requirements, low staffing levels have been observed in NICUs in North America and Europe and further, low staffing levels have been associated with poor neonatal outcomes (e.g., mortality, nosocomial infection, intracranial hemorrhage) [12–14]. In earlier studies, nurses have reported that care rationing is highly prevalent in this setting, with the most frequently rationed nursing interventions being discharge preparation and infant comfort care [11, 15]. The rationing of infant comfort care is particularly worrisome given the documented effectiveness of nursing interventions to address neonatal pain and the critical mass of evidence suggesting that adequate neonatal pain management is associated with improved developmental and biobehavioral outcomes in later life [16–18]. Similarly, the rationing of discharge preparation is also alarming given that decreased readiness for discharge has been associated with poorer infant and parent outcomes (e.g., anxiety and coping difficulties), as well as greater post-discharge health services utilization [19, 20]. However, to the best of our knowledge, no previous study has examined whether the rationing of discharge preparation and infant comfort care are associated with lack of readiness for hospital discharge and poorer neonatal pain control, respectively. The purpose of this study was to examine these relationships.

### Conceptual framework

At least two conceptual frameworks have been proposed to explain the process of nursing care rationing [6, 21]. These frameworks are both based on Donabedian’s structure-process-outcome model [22]. Accordingly, they both conceptualize the rationing of nursing care as a process-oriented measure of healthcare quality. Within these models, care rationing is hypothesized to occur as a response to hospital structural contingencies (e.g., understaffing, non-supportive work environments, lack of resources) and to be influenced by nurse characteristics (e.g., education, experience, decision-making skills), as well as by patient requirements for nursing care and severity of illness [7, 8]. In turn, care rationing is presumed to result in potentially preventable adverse outcomes. While considerable research work has been conducted on the structural determinants of care rationing [5, 9, 23], comparatively little attention has been given to whether the rationing of specific nursing interventions (e.g., discharge preparation, comfort care) is associated with poorer patient outcomes that are clinically connected to them (e.g., lack of readiness for hospital discharge, poor pain control) [5].

## Methods

### Design and study population

A cross-sectional mail survey was conducted in late 2014. The population for this study included RNs who worked in NICUs in the Canadian province of Quebec. Specifically, to be eligible for the study, RNs needed to: a) hold an active professional nursing license in Quebec, b) report working in a NICU at the time of their annual license renewal and, c) have agreed to the release of their mailing address to researchers by the Quebec Order of Nurses. According to statistics from the Quebec Order of Nurses, in 2014, 720 RNs reported working in one of seven NICUs in the province, of whom 285 (39.6 %) agreed to be contacted by mail for research purposes.

### Recruitment

Each potential participant received a package by mail containing an introduction letter, a prepaid return envelope, and the French or English version of the survey, depending on the language they use to communicate with their licensing body. The survey included validated instruments measuring care rationing, parent and infant readiness for discharge, neonatal pain control, and a series of questions on RNs’ demographic, professional, and employment characteristics. Non-respondents were sent a maximum of two reminder letters at two and four weeks following the initial contact [24]. Data collection occurred over a six-week period in November and December 2014. Based on sample size calculations, a minimum of 100 surveys was required to detect an effect size (f^2^) of 0.15 at a Type 1 error rate (α) of 5 %, assuming power (β) of 80 %. To assess the accuracy of the data entry, validity checks were performed on a random 10 % of the data. No data entry errors were identified.

### Measures

#### Independent variables

The rationing of neonatal nursing care interventions was measured using the Neonatal Extent of Work Rationing Instrument (NEWRI) [11]. The NEWRI is composed of 59 items. For each item, RNs are asked to indicate on a 4-point Likert-type scale (ranging from 1 = very rarely to 4 = very often) the frequency with which they rationed the stated nursing interventions for lack of time or resources over the past 30 days (e.g., “assess patient signs and symptoms”, “support, assist or encourage parents in performing infant's care”).

Content validity of both the French and English versions of the NEWRI was established through consultation with an expert panel of ten bilingual clinical nurse specialists in neonatal nursing holding a Master’s or higher degree, all of whom had a minimum of two years of clinical experience in neonatal nursing [11]. Factor analysis of the items on the NEWRI identified four subscales: (a) Life Support and Technology-Oriented Nursing Care (15 items), (b) Parental Support and Teaching and Infant Comfort Care (12 items), (c) Patient Surveillance (seven items), and (d) Discharge Preparation (six items); with Cronbach’s alphas for these subscales ranging from 0.81 to 0.93 [11]. The mean item score on each of the four subscales was computed. In the analyses, the two independent variables of interest were Parental Support and Teaching and Infant Comfort Care and Discharge Preparation, whereas scores on Life Support and Technology-Oriented Nursing Care and Patient Surveillance subscales were used as controls.

#### Dependent variables

##### Readiness for hospital discharge

Readiness for discharge is defined as an estimate of the patients’ and families’ ability to leave the care facility, a perception of being prepared for hospital discharge, and an indicator of sufficient recovery to allow safe discharge [19, 25]. Parent and infant readiness for NICU discharge can be assessed by either the nurses, the physicians or the parents themselves [26]. However, nurses’ assessments have been found to be better predictors of parent and infant future needs for healthcare services than the readiness assessments of physicians and parents [19, 27]. Therefore, we measured nurses’ perceptions of parent and infant readiness for hospital discharge using the Readiness for Hospital Discharge Scale – Nurse Form (RHDS – Nurse Form) [20]. The RHDS – Nurse Form contains 29 items subdivided into 5 subscales: 1) parental personal status (8 items), 2) child personal status (5 items), 3) knowledge (9 items), 4) coping ability (3 items), and 5) expected support (4 items) [20]. The RHDS – Nurse Form uses an 11-point scaling format with anchor words (e.g., not ready, totally ready) placed at the 0 and 10 poles of the scale [26]. For the purpose of this study, RNs were asked to report on the level of readiness for NICU discharge of parents and infants on their units over the past 30 days. In the analyses, the overall mean score on the RHDS – Nurse Form (i.e., the mean score of all subscales) was used as the dependent variable. The reliability, construct validity, and predictive validity of the English version of the RHDS have been established in several studies [25–27]. The Cronbach’s alphas for different forms of the RHDS have been shown to range from 0.82 to 0.90 [20, 26, 28]. In addition, the reliability and validity of the French version of the instrument have also been established (M. Weiss, personal communication, January 21, 2014).

##### Neonatal pain control

NICU nurses’ perceptions of neonatal pain control were assessed using a single item chosen from the Neonatal Nurses’ Perceptions of Pain Assessment and Management in NICUs Survey [29]. This item asked nurses to rate their overall perception of infant pain control on their unit over the past month (i.e., “I feel that neonatal pain on my unit is well managed”) [29]. This item, translated from English to French for the purposes of this study, was measured on a 5-point Likert-type scale (ranging from 1 = strongly agree to 5 = strongly disagree). Prior studies have provided evidence that nurses’ assessments are an accurate indicator of neonatal pain control [30, 31]. Moreover, researchers have repeatedly demonstrated the reliability and validity of single-item measures designed to capture global constructs such as pain control [32, 33].

### Confounding variables

Prior studies have shown that nurses’ characteristics can influence perceptions of care rationing, parents and infants’ readiness for discharge, and neonatal pain control [6, 21, 34]. For this reason, the survey included a series of questions regarding respondents’ demographic characteristics (i.e., age, sex, race), professional background (i.e., highest educational attainment, years of experience) and nature of employment (i.e., full-time vs. part-time, and permanent vs. temporary).

### Data analyses

Descriptive statistics were used to summarize the study variables. Given low rates of missing data, missing values were imputed using the mean score for the particular variable. Multivariable linear regression was used to examine the association between care rationing and each of the two study outcomes analysed as continuous variables: 1) parent and infant readiness for NICU discharge and, 2) neonatal pain control. A separate regression model was fitted for each outcome. These models used two subscale scores on the NEWRI as independent variables (i.e., Parental Support and Teaching and Infant Comfort Care and Discharge Preparation), while controlling for nurses’ demographic, professional and employment characteristics and the rationing of Life Support and Technology-Oriented Nursing Care and Patient Surveillance. To account for the effect of nurse clustering within a given NICU, regression models were fitted using the generalized estimating equation (GEE) framework [35]. To account for the small number of clusters (*n* = 7 NICUs), standard error estimates were corrected using a procedure described by Morel [36, 37].

To facilitate interpretation of the regression coefficients, effect sizes were computed in the form of percentage changes in the dependent variables associated with a one-unit increase in the independent variable. To determine these percentages, regression coefficients were first divided by the maximum range of change possible for a given scale (e.g., a four-point Likert-type scale with scores ranging from 1 to 4 has a possibility of three units of change, i.e., from 1 to 2, 2 to 3 and 3 to 4). These quotients were then multiplied by 100 to yield percentages [11]. Statistical analyses were conducted using SAS version 9.3, and *p* < 0.05 was used as the criterion to assess statistical significance.

## Results

A total of 285 NICU RNs were contacted for the purpose of this study, and 125 returned a completed survey; resulting in a response rate of 44.0 %. All but five RNs completed the survey in French and the rate of missing values per variable was low (Range: 0–5.6 %). The typical participant was a Caucasian female, aged between 26 and 30 years (Table 1). She had received her initial nursing education in a three-year diploma program, and had gone on to complete a bachelor’s degree (Table 1). The average participant also held a part-time position and had 11 years of nursing experience, nine of which were in neonatal nursing (Table 1).

**Table 1:** Characteristics of the participants (*N* = 125)

Demographic characteristics	
Sex	
Female – *n* (%)	125 (100.0)
Race	
Caucasian – *n* (%)	112 (89 · 6)
Age – *n* (%)	
Less than 20 years	0 (0.0)
20–30 years	60 (48 · 0)
31–40 years	37 (29 · 6)
41–50 years	11 (8 · 8)
51–60 years	15 (12 · 0)
61 years and above	2 (1 · 6)
Professional characteristics	
Initial nursing education - *n* (%)	
Hospital diploma	1 (0 · 8)
College diploma	95 (76 · 0)
Baccalaureate degree	28 (22 · 4)
Master’s degree and above	1 (0 · 8)
Highest degree currently held – *n* (%)	
Hospital diploma	0 (0.0)
College diploma	58 (46 · 4)
Baccalaureate degree	61 (48 · 8)
Master’s degree and above	6 (4 · 8)
Years of experience	
As a nurse (M ± SD)	11 · 1 ± 10 · 0
At current hospital (M ± SD)	10 · 7 ± 9 · 9
In neonatal care (M ± SD)	9 · 2 ± 9 · 2
Type of nursing position currently held	
Full-time – *n* (%)	59 (47 · 2)
Part-time – *n* (%)	66 (52 · 8)

*Abbreviations*: *M* mean, *SD* Standard deviation

Mean values for the independent and dependent variables used in the regression models are listed in Table 2. To facilitate interpretation of these values, some additional statistics are provided. Indeed, using the scale mid-point as the criterion on the care rationing subscales (i.e., the value indicating neither very often nor very rarely), we observed that 40.0 % of the respondents reported rationing Discharge Preparation often or very often, while 28.0 % reported rationing Parental Support and Teaching and Infant Comfort care often or very often. In comparison, only 7.2 and 9.6 % of these nurses, respectively, reported rationing Life Support and Technology-Oriented Nursing Care and Patient Surveillance often or very often.

**Table 2:** Descriptive statistics: independent and dependent variables (*n* = 125)

Independent variables	Mean ± SD
Care Rationing (NEWRI)^a^	
Life support and technology-oriented nursing care	1 · 53 ± 0 · 57
Patient surveillance	1 · 67 ± 0 · 69
Parental teaching, support, and infant comfort care	2 · 20 ± 0 · 59
Discharge preparation	2 · 33 ± 0 · 59
Dependent variables	
Readiness for hospital discharge (RHDS)^b^ – overall score	6 · 81 ± 0 · 93
Neonatal pain control^c^	2 · 91 ± 1 · 06

*Abbreviations*: *NEWRI* Neonatal Extent of Work Rationing Instrument, *RHDS* Readiness for Hospital Discharge Scale – Nurse Form, *SD* standard deviation

^a^Scores on the NEWRI’s subscales range from 1 (very rarely) to 4 (very often); ^b^Scores on the RHDS – Nurse Form range from 0 (not ready) to 11 (totally ready); ^c^Neonatal pain control was measured using a single-item scale asking nurses to rate their overall perception that neonatal pain was well managed on their unit over the past month. This scale ranged from 0 (strongly agree) to 5 (strongly disagree)

In addition, 15.2 % of the respondents felt that parents and infants were not well prepared for NICU discharge. Furthermore, using mean scores above 2.5 on the single item measuring nurses’ perceptions of pain management in the NICU as a criterion (i.e., the value indicating a ‘neutral’ opinion about the quality of pain management), we observed that 54.4 % of RNs reported that pain had not been well managed on their unit in the past month.

### Multivariate analysis

In the regression analyses, after adjusting for nurses’ demographic, professional and employment characteristics, we found that RNs’ increased perceptions of rationing of Discharge Preparation was significantly related to worse perceptions of parent and infant readiness for NICU discharge (Table 3). Specifically, every one-point increase in the rationing of Discharge Preparation was associated with a 4.8 % decrease in overall readiness for NICU discharge score (i.e., −0.53/11 units = −0.048 or −4.8 %) (Table 3). Similarly, reports of rationing of Parental Support and Teaching and Infant Comfort Care were significantly and inversely related to nurses’ perceptions of readiness for NICU discharge (Table 3). The observed regression coefficient suggests that every one-point increase in the rationing of Parental Support and Teaching and Infant Comfort Care is associated with a 4.1 % reduction in overall readiness for NICU discharge (i.e., −0.46/11 units = −0.041 or −4.1 %) (Table 3). Lastly, we observed that the rationing of Parental Support and Teaching and Infant Comfort care was statistically significantly related to decreased levels of perceived neonatal pain control (Table 3). Specifically, a one-unit increase in the rationing of Parental Support and Teaching and Infant Comfort Care was related to a 19.2 % reduction in nurses’ confidence that neonatal pain was well managed on their unit over the previous month (i.e., 0.96/5 = 0.192 or 19.2 %).

**Table 3:** Fully adjusted regression models^a^ of the effects of care rationing on readiness for discharge and pain control (*n* = 125)

Care rationing (NEWRI)	Overall readiness for NICU discharge Estimate (95 % CI)	Pain control Estimate (95 % CI)
Parental support and teaching and infant comfort care	−0 · 46 (−0 · 73; −0 · 20)^**^	0 · 96 (0 · 41; 1 · 50)^**^
Discharge preparation	−0 · 53 (−0 · 71; −0 · 35)^**^	−0 · 01 (−0 · 38; 0 · 36)

*Abbreviations*: *NEWRI* Neonatal Extent of Work Rationing Instrument, *NICU* neonatal intensive care unit

**p < 0 · 01

^a^Regression coefficients are from models using the generalized estimating equation (GEE) framework to adjust for the effect on nurse clustering within a given NICU. To control for the inflation of Type 1 error associated with performing GEE on a small number of clusters (*n* = 7 NICUs), the modified GEE approach proposed by Morel et al. [36] was used. The adjusted models used the four subscales scores on the NEWRI as independent variables while controlling for nurses’ professional, demographic and employment characteristics, including: number of years worked as a nurse, highest degree actually held (college and hospital diploma vs. baccalaureate degree and above), full- vs. part-time employment, race (Caucasian vs. other)

## Discussion

The purpose of this study was to examine whether the rationing of discharge preparation and infant comfort care, the two most frequently rationed neonatal nursing interventions [11, 15], were associated with lack of readiness for NICU discharge and poorer neonatal pain control, respectively. In both cases, we found evidence for these associations.

We found that RNs’ perceptions of increased rationing of Discharge Preparation and of Parental Support and Teaching were both independently and significantly related to their perceptions of lower parent and infant readiness for NICU discharge. Recent studies suggest that understaffing, high patient census and turnover and non-supportive work environments (e.g., high non-nursing task requirements) all compete with NICU nurses’ time for teaching and discharge preparation [15, 19, 38–40]. In addition, researchers have found that mothers who are unprepared for NICU discharge are more likely to report difficulty coping with infant care at home, adopt potentially unhealthy infant care behaviors, express a greater number of physical and psychosocial issues or complications, and require more unscheduled visits to healthcare providers in the first months following discharge [19, 27, 40, 41]. Our results therefore add to this emerging body of literature by suggesting that when NICU nurses perceive they do not have sufficient time and resources, they will consequently ration important nursing interventions that are required to adequately prepare parents and infants for NICU discharge. Future research should examine whether the rationing of Discharge Preparation and of Parental Support and Teaching is associated with increased occurrence of independently measured adverse post-discharge outcomes.

The second finding of this study was the relationship of higher rationing of Parental Support and Teaching and Infant Comfort Care with decreased levels of perceived pain control. Parental support and teaching in the NICU involves instructing parents on a variety of comfort measures, such as kangaroo care and breastfeeding, which have been observed to have analgesic effects [16, 17]. NICU nurses can similarly use a variety of nonpharmacological (e.g., swaddling, non-nutritive sucking) and pharmacological interventions (e.g., using sucrose or other analgesic medications) to ease neonatal pain, stress and discomfort [17]. Consistent with prior research [34, 42], we found that lack of time and resources in the NICU act as barriers to the effective application of pain control measures by nurses, which presumably leads to less favourable evaluations of the quality of pain management. Given that numerous studies have documented the adverse consequences of poor pain management during the neonatal period on later developmental and biobehavioral outcomes [18], there is a pressing need to further examine the relationships between care rationing and scores on standardized pain assessment scales and physiological indices of pain in NICU patients.

Moreover, using the NEWRI, we were able to quantify the extent of rationing of neonatal nursing interventions in Quebec’s NICUs. As can be noted in the following tabulation, the frequency with which neonatal nursing interventions are rationed in Quebec’s NICUs appears to have worsened since our previous investigation using the NEWRI in this same population of nurses [11]:

**Table:** 

Neonatal Nursing Interventions	Extent of Rationing in Quebec’s NICUs	
	2010	2015 (present study)
Life support and technology-oriented nursing care	0.9 %	7.2 %
Patient surveillance	5.9 %	9.6 %
Parental teaching, support, and infant comfort care	20.1 %	28.0 %
Discharge preparation	28.1 %	40.0 %

To our knowledge, these represent the first longitudinal data on the variations in the extent of rationing of nursing care interventions in a given population through time. While these data are based on two time points, they nonetheless suggest that the conditions which lead to the rationing of these nursing interventions (e.g., understaffing and non-supportive work environment) may have further deteriorated in the 5-year period that separates the two studies; a hypothesis that warrants further investigation.

Similar to our earlier findings [11], we again noted that Discharge Preparation and Parental Support and Teaching and Infant Comfort Care were more frequently rationed than Life Support and Technology-Oriented Nursing Care interventions or Patient Surveillance. This pattern is also consistent with several recent reviews of studies conducted in other clinical settings, patient populations and jurisdictions [5, 9, 23], as well as with the results of a small study conducted in USA NICUs [15]. Overall, this pattern suggests that in the face of limited resources, NICU nurses prioritize potentially life-saving interventions (e.g., patient surveillance and technology-oriented care) over less critically important ones such as discharge planning or parental support and teaching and infant comfort care. While such decisions are potentially beneficial for patient safety, they may not be without consequences for the infants and their parents.

Indeed, we found that 15.2 % of surveyed RNs felt that infants and parents were underprepared for NICU discharge, and 54.4 % believed that pain was not well managed on their unit. These findings, which are in agreement with previous reports by NICU nurses from other countries [42–44], are particularly worrisome given the aforementioned adverse consequences associated with a lack of readiness for NICU discharge and uncontrolled neonatal pain.

Several limitations of this study should be acknowledged. First, our results may suffer from non-response bias. Indeed, while our response rate of 44.0 % compares favorably to those observed in recent mail surveys of RNs’ perceptions of the rationing of nursing care interventions [5, 9], our overall sampling frame was limited by the small proportion of NICU nurses in the province of Quebec (39 %) who had consented to the release of their mailing addresses to researchers. However, a comparison of the demographic characteristics of the respondents and non-respondents (including those not agreeing to the release of their mailing address) suggests that our sample was representative of the population of NICU nurses in the province of Quebec (data not shown). Second, as is the case with nearly all studies in this area of research [5], both the independent and dependent variables were based on NICU nurses’ perceptions. As a consequence, it is possible that nurses who have more unfavorable perceptions about care rationing may also believe that parent and infant readiness for discharge and neonatal pain control are suboptimal on their unit, when this may not, in fact, be the reality. Similarly, it is possible that nurses’ perceptions could be influenced by a variety of unmeasured factors, such as additional specialized training obtained by the nurses (e.g., developmental care certification) or the availability of dedicated discharge teams or pain management consultants in the NICU environment. Future studies examining the associations between the rationing of specific nursing interventions and patient outcomes should measure and potentially control for such factors. Lastly, cross-sectional analyses cannot provide definitive evidence for causal relationships. Longitudinal studies, including intervention trials, are thus needed to determine how the antecedent exposure to the rationing of nursing care interventions may influence both in-hospital and post-NICU discharge outcomes. Despite the aforementioned limitations, our findings are consistent with trends and patterns observed in other studies conducted in a variety of patient populations worldwide; which lends credibility to the results of this study.

## Conclusion

We found that the rationing of Discharge Preparation and Parental Support and Teaching and Infant Comfort care is related to NICU nurses’ perceptions of decreased parent and infant readiness for NICU discharge. In addition, we observed that the rationing of Parental Support and Teaching and Infant Comfort care is also related to decreased levels of perceived neonatal pain control. These results further emphasize the relevance of nursing care rationing for patient safety and quality of care in the NICU while simultaneously adding to the growing body of evidence validating the concept of care rationing across nursing specialities. The results of this study also highlight the necessity for hospital managers to intervene on the potentially modifiable determinants of care rationing (e.g., understaffing, poor work environments, and lack of resources and support for nursing care). Additional studies are needed to further document the impact of care rationing on objective pain assessments in neonates as well as on post-NICU discharge outcomes. Similarly, investigations of the connections between the rationing of specific nursing care interventions and patient outcomes in other clinical populations and health care systems should continue.

## Abbreviations

GEE, generalized estimating equation; NEWRI, Neonatal Extent of Work Rationing Instrument; NICU, neonatal intensive care unit; RN: registered nurse; RHDS, Readiness for Hospital Discharge Scale
